# A Highly Spatiotemporal Resolved Pyrometry for Combustion Temperature Measurement of Single Microparticles Applied in Powder-Fueled Ramjets

**DOI:** 10.3390/nano15030223

**Published:** 2025-01-30

**Authors:** Zhangtao Wang, Xunjie Lin, Xuefeng Huang, Houye Huang, Minqi Zhang, Qinnan Yu, Chao Cui, Shengji Li

**Affiliations:** 1 Institute of Energy, Department of Physics, Hangzhou Dianzi University, Hangzhou 310018, China; wangzhangtao429@outlook.com (Z.W.); lin891@hdu.edu.cn (X.L.); 15108404571@163.com (H.H.); 2142010021@hdu.edu.cn (M.Z.); wsplxjsh@outlook.com (Q.Y.); 2 Zhejiang Institute of Quality Sciences, Hangzhou 310018, China; ccpp208@163.com; 3 College of Materials and Environmental Engineering, Hangzhou Dianzi University, Hangzhou 310018, China

**Keywords:** combustion temperature measurement, pyrometry, metal fuel particles, laser ignition, powder-fueled ramjets

## Abstract

It is vital to measure combustion temperature to define combustion models accurately. For single fuel particles in powder-fueled ramjets, their size distribution ranges from submicron to submillimeter, and their burn time is short to millisecond order. Moreover, the radiation intensity of different types of fuel particles significantly oscillated with several orders of magnitude. Current temperature measurement technology is facing this challenge. This paper proposes a highly spatiotemporal resolved pyrometry to measure the combustion temperature of fuel particles by coupling single-point photomultiplier tube (PMT)-based and two-dimensional complementary metal oxide semiconductor (CMOS)-based photoelectric devices. Both the offline calibration by blackbody furnace and online calibration by standard lamp confirmed the measurement accuracy of the pyrometry. Then, the pyrometry was used to measure the combustion temperature of fuel particles including micro-Al, nano-Al, micro-Mg, nano-B, and micro-B_4_C. The temperature evolution and distribution of burning fuel particles were complementarily obtained, especially the interfacial flame temperature near the particle surface. Based on the obtained combustion temperature, the combustion characteristics and the energy release efficiencies among these fuels were evaluated and compared in detail, which are helpful to recognize, in depth, the combustion behavior and reveal the combustion mechanism of fuel particles in powder-fueled ramjets.

## 1. Introduction

With the development of the ramjet, hypersonic vehicles with high Mach numbers and wide domain requirements have been widely considered [1,2,3]. Traditionally, gas- (like hydrogen/ethylene) [4,5,6] or liquid (jet fuels such as kerosene, JP-10, etc.) [7,8,9]-fueled ramjets are the primary forms of power engines for hypersonic vehicles, especially liquid-fueled ramjets. Nevertheless, at high Mach numbers, liquid-fueled ramjets encountered a bottleneck into the practical engineering application due to the low density of liquid fuel, high dissociation, poor combustion stability, and complex engine structure [10,11,12]. Therefore, solid-fueled ramjets have gradually become an interesting and competitive choice, since there are the advantages of relatively simple structures, lightened weight, high density-specific impulse, low cost, good security, and short response time over liquid-fueled ramjets [13,14]. In particular, powder-fueled ramjets have drawn much attention in recent years [15], owing to high safety and stability during manufacture, storage, and use, and the function of re-ignition and adjustable thrusts via flow rate regulation [16]. More importantly, powder fuels possess low dissociation property and keep full energy release even in the flight state at high Mach numbers [17].

In powder-fueled ramjets, commonly employed powder fuels include Al, Mg, B, composite fuel, etc. [18,19,20,21,22]. Xia et al. [23] reported an Al-based powder-fueled ramjet and experimentally achieved ignition and self-sustaining combustion behavior and found a severe combustion deposition phenomenon. Kong et al. [24] designed a standing vortex Mg-based powder-fueled ramjet with the primary and secondary air intake modes. The ignition, combustion deposition, and combustion efficiency were numerically simulated. Yang et al. [25,26,27] also demonstrated a Mg-based powder-fueled ramjet with one-dimensional Mg dust cloud laminar flow combustion for self-sustained stable combustion technology. Dong et al. [28] proposed a new ramjet engine configuration based on a solid/powder fuel combination, which was fueled by B powder particles and adopted a secondary air intake. Regardless of the type of powder fuel, a comprehensive understanding of the energy release and thermodynamic state of powder fuel particles via combustion is crucial to improve the specific impulse performance of powder-fueled ramjets.

The combustion temperature is one of the most important parameters to characterize the energy release and thermodynamic state during the combustion of powder fuel in the ramjets, which is imperative to accurately build the computational combustion models [29,30,31]. In recent decades, the combustion temperatures of employed powder fuel particles have been widely investigated via several techniques based on the radiation methodology and principle, including single-point spectroscopy [32,33], single-point pyrometry [34,35,36], laser interferometry and holography [37], coherent anti-Stokes Raman spectroscopy (CARS) [38,39,40], etc. The temporal resolution of single-point spectroscopy and CARS, typically kHz level due to spectrometer limitations, is inadequate for capturing the complete combustion temperature evolution of micro/nano fuel particles. Single-point photometric methods, though highly temporally resolved, provide only one-dimensional temperature data, precluding detailed particle temperature trajectory analysis during combustion. However, highly spatiotemporal-resolved temperature distribution measurements of employed powder fuel particles in the ramjets have been sparsely investigated. For single fuel particles in powder-fueled ramjets, their size distribution ranges from submicron to submillimeter, and the corresponding burn time is rather short to millisecond order. Furthermore, the radiation intensity of different types of fuel particles during combustion significantly oscillated with several orders of magnitude, resulting in an extremely weak or overexposed signal response in the photoelectric detectors and failure in the temperature measurement.

In response to these issues, this work proposed, designed, and manufactured a coupled PMT&CMOS pyrometry (coupling single-point PMT-based and spatially resolved CMOS-based photoelectric devices) with the high temporal and spatial resolution of microsecond and submicron, which could meet the combustion temperature measuring requirements of multiple fuel particles applied in powder-fueled ramjets. The PMT-based and CMOS-based pyrometry was calibrated offline by the blackbody furnace, respectively. Furthermore, the coupled PMT&CMOS pyrometry was installed into the experimental setup for characterizing the ignition and combustion characteristics of single fuel particles and was further online calibrated by the standard lamp. In addition, the combustion temperature of micrometer-sized aluminum (micro-Al), magnesium (micro-Mg), boron carbide (micro-B_4_C), nanometer-sized aluminum (nano-Al), and boron (nano-B) fuel particles were measured, and the results were deeply analyzed and discussed. The comparisons between different types of fuels were made to evaluate the combustion characteristics of metallic fuel particles and the modification performance of composite fuel or alloy fuel particles.

## 2. Methodology

### 2.1. Materials

In this work, five types of fuel particles were chosen to conduct the combustion test, including micro-Al, nano-Al, micro-Mg, nano-B, and micro-B_4_C. Micro-Al powder was obtained from Xi’an Modern Chemistry Research Institute (Xi’an, China), which was fabricated by the gas atomization method, and the purity was as high as 99.9%. The particle size ranged from ~5.0 μm to 70.0 μm and the median size was ~29.0 μm. Nano-Al powder was purchased from Dk Nano Technology Co., Ltd. (Beijing, China). The purity was ~99.9%, and the nominal particle size was ~50 nm. Its density and specific surface area were 0.23 g/cm^3^ and 20 m^2^/g, respectively. Micro-Mg powder was purchased from Xiao Huang Nanotechnology Co., Ltd., Shanghai, China, which was also prepared by the gas atomization method, and the purity was over 99.9%. Its median size was ~40.0 μm. Nano-B powder was also purchased from Dk Nano Technology Co., Ltd. (Beijing, China). The purity reached 99.9%. The nominal particle size was ~100 nm, and the specific surface area was 49 m^2^/g. Micro-B_4_C powder was obtained from the Institute of Metal Research Chinese Academy of Sciences (Shenyang, China), whose purity was over 99.9%. The size range of particles was 15.0 μm ~30.0 μm, and the median size was ~25.0 μm.

### 2.2. Experimental Setup for Ignition and Combustion Characterization

**Figure 1:**
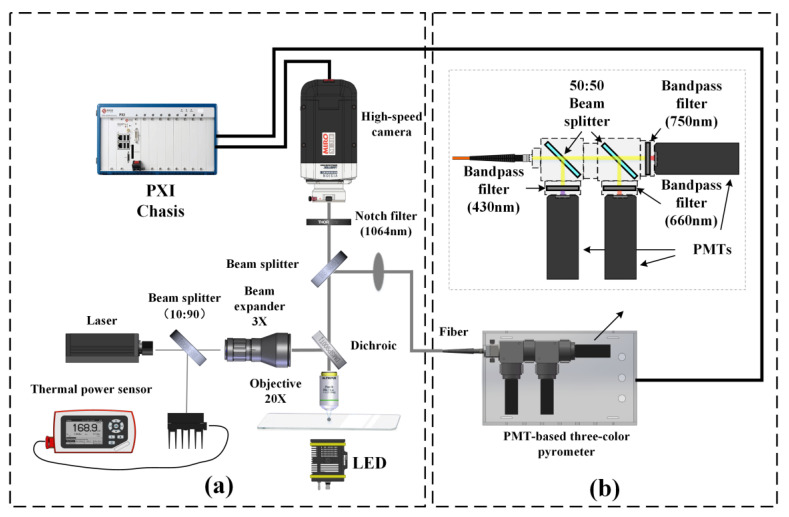
Schematic of experimental setup for ignition and combustion characterization (**a**) and PMT-based three-color pyrometry (**b**).

Figure 1a shows the schematic of the experimental setup, which was designed and constructed to ignite all kinds of individual powder fuel particles and measure the combustion temperature. The experimental setup mainly includes the laser ignition module, the coupled pyrometry module, and the synchronous triggering and data acquisition module. For the laser ignition module, a continuous Nd: YVO_4_ laser (TEM_00_ mode, wavelength of 1064 nm, and power of 0~2 W) was selected as the ignition source. The laser beam routed the beam splitter 1 (10:90) and a beam expander, then transmitted through a dichroic, and finally focused on a single isolated fuel particle by a microscopic objective (20×) to ignite. The focused beam diameter was ~18.8 μm. The ignition power density ranged from 0 to 7.21 × 10^5^ W/cm^2^, which could be precisely controlled by adjusting laser output power. More details on the ignition module can be found in our previous work [41,42,43].

The coupled pyrometry module is composed of PMT-based pyrometry, CMOS-based pyrometry, and receiving components. The radiation signal of a single isolated fuel particle was collected by the microscopic objective and transmitted through the dichroic and the beam splitter (50:50) into the PMT-based pyrometry and CMOS-based pyrometry, respectively. The PMT-based pyrometry consists of three PMTs (H10722, Hamamatsu, Japan), two beam splitters, and three 10 nm bandpass filters with the central wavelengths of 430 nm, 660 nm, and 750 nm, as shown in Figure 1b. The temporal resolution of PMT-based pyrometry was kept at 50 μs. The CMOS-based pyrometry includes a 10 nm notch filter with a central wavelength of 1064 nm and a high-speed CMOS camera. The temporal resolution of CMOS-based pyrometry depends on the shooting frames and exposure time. In this work, the exposure times of 5 μs (micro-Mg), 50 μs (micro-B_4_C), 90 μs (nano-Al), or 99 μs (micro-Al and nano-B) were set to follow the pace of the PMT-based pyrometry. The synchronous triggering and data acquisition module was constructed via the PXI (PCI extensions for Instrumentation) bus technology, which enabled us to synchronously control the laser, PMTs, and high-speed CMOS camera.

### 2.3. Calibration of Coupled PMT&CMOS Pyrometry

The calibration of coupled PMT&CMOS pyrometry followed two steps. The first one was the offline calibration of independent PMT-based pyrometry or CMOS-based pyrometry by using a blackbody furnace. The second one was the online calibration of coupled PMT&CMOS pyrometry installed into the ignition apparatus via a standard lamp.

#### 2.3.1. Offline Calibration

**Figure 2:**
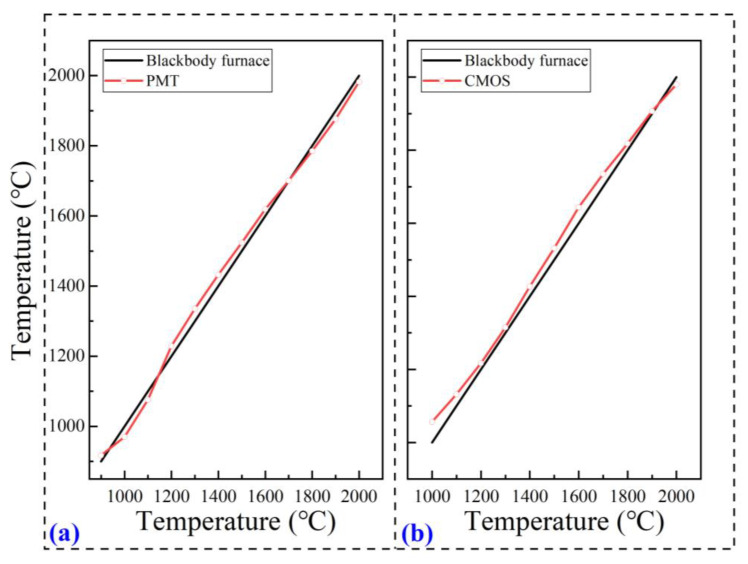
The offline calibrated temperature curves of independent PMT-based pyrometry (**a**) and CMOS-based pyrometry (**b**) via a blackbody furnace.

The spectral response coefficient of PMT-based pyrometry can be determined by comparing the ratio of the blackbody furnace radiation spectral signals with the actual response voltage values of PMT-based pyrometry. To ensure calibration accuracy, the same control voltage was used to eliminate the influence of the internal amplification module of the PMTs on the measurement. The blackbody furnace (Mikron M390, Santa Barbara, CA, USA) was set to the most suitable temperature of 1700 °C and operated for 15 min, since the radiation signal intensity of the blackbody furnace at this temperature can meet the requirement of PMT response with the low supply voltage and high signal-to-noise ratio. The entire calibration process was conducted in total darkness to minimize the influence of background light on the test results. The offline calibration of independent PMT-based pyrometry is shown in Figure 2a, in the range of 900 °C~2000 °C with an interval of 100 °C. It illustrates excellent linearity and a low relative error of 2.75%, suggesting that the temperature measured by PMT-based pyrometry had good agreement with the set temperature of the blackbody furnace.

Similarly, the offline calibration of CMOS-based pyrometry via the blackbody furnace is shown in Figure 2b. For this calibration, the average temperature of the two-dimensional temperature distribution was calculated and compared with the set temperature. Obviously, the largest deviation was 5.7% in the occurrence at the blackbody furnace temperature of 1000 °C, which is acceptable for the measurement of high temperature.

#### 2.3.2. Online Calibration

**Figure 3:**
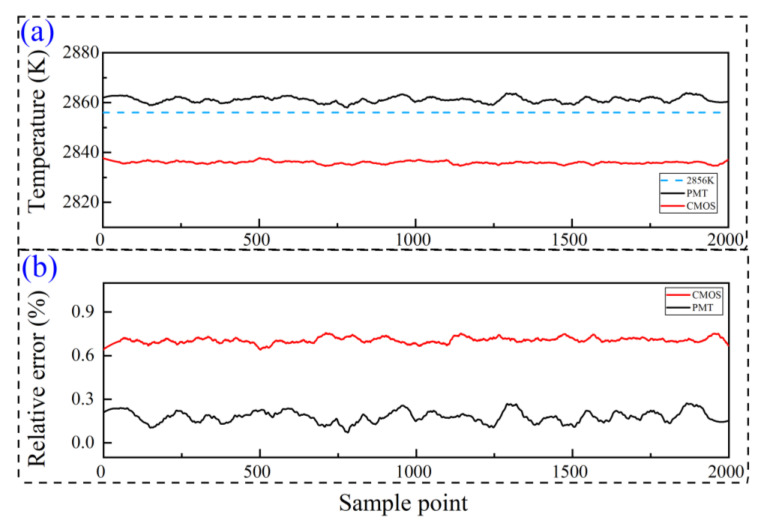
The online calibrated temperature curves of coupled PMT&CMOS pyrometry (**a**) and measurement errors (**b**) via a standard lamp with 2856 K color temperature.

The online calibration of coupled PMT&CMOS pyrometry equipped into the ignition system was conducted via a standard lamp with 2856 K color temperature. The calibration was used to eliminate the influence of additional optical components into the coupled pyrometry on the temperature measurement. During the illumination of the standard lamp for 5 s, the calibrated curves were illustrated in Figure 3a. It demonstrated that the average PMT-based and CMOS-based temperatures were 2860 K and 2838 K, respectively. The measured temperature slightly oscillated around the average value with the sample point. Figure 3b shows the measurement errors of coupled PMT&CMOS pyrometry, and the maximum measurement errors of PMT-based and CMOS-based temperatures were 0.45% and 0.76%, respectively. It is worth noting that all the PMT-based temperatures were slightly higher than 2856 K color temperature, suggesting that the measured temperature was possibly overestimated. On the contrary, all the CMOS-based temperatures were lower than 2856 K color temperature with an underestimation. The slight overestimation and underestimation mainly result from the influence of the band width of filters and the depth of field of the receiving optical microscopic objective.

## 3. Results and Discussion

### 3.1. Combustion Temperature Measurement of Al Fuel Particles

There are significant differences between micro-Al and nano-Al powders in the application of powder-fueled ramjets, which mainly include combustion characteristics, conveying characteristics, and injection efficiency. In terms of combustion characteristics, Al powders with different sizes may undergo different phase transition phenomena such as melting and boiling during the reaction with oxygen, resulting in different combustion temperature and combustion time and significant fluctuations in burning rate [44,45]. Therefore, to evaluate the effect of Al particle size on the combustion temperature, in this work, micro-Al and nano-Al particles (50 nm) were selected to conduct the comparative experiment.

#### 3.1.1. Combustion Temperature Measurement of Micro-Al Fuel Particles

**Figure 4:**
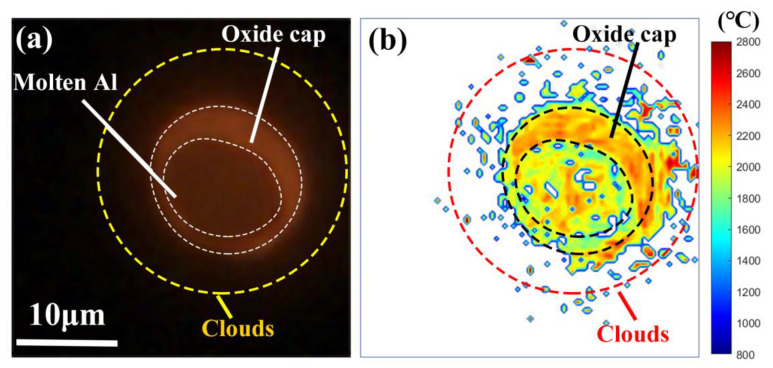
(**a**) A snapshot of a two-dimensional flame image of a single isolated micro-Al particle burned in air and (**b**) corresponding two-dimensional temperature distribution via coupled PMT&CMOS pyrometry.

Figure 4a shows a two-dimensional flame image of a single isolated micro-Al (the equivalent diameter of 15.3 μm) burned in air at atmospheric pressure, and accordingly, the two-dimensional temperature distribution via coupled PMT&CMOS pyrometry is shown in Figure 4b. In Figure 4a, the image clearly demonstrated the molten aluminum, the oxide cap, and the clouds due to the uniform emissivities and radiation luminance of these matters in the field of view [46]. It can be identified that from the two-dimensional temperature distribution in Figure 4b, the temperature of the oxide cap reached 2000 °C and above, and the temperature of the molten aluminum was further lower than 1500 °C; however, the temperature of the clouds was within the temperature range of both. The cloud zones around the oxide appeared to show low emission and luminance, which possibly resulted from condensed small Al oxide smoke particles. The estimated cloud temperature may be less accurate since they can be optically thin and integrated through the flame depth and biased toward the highest temperatures [47].

**Figure 5:**
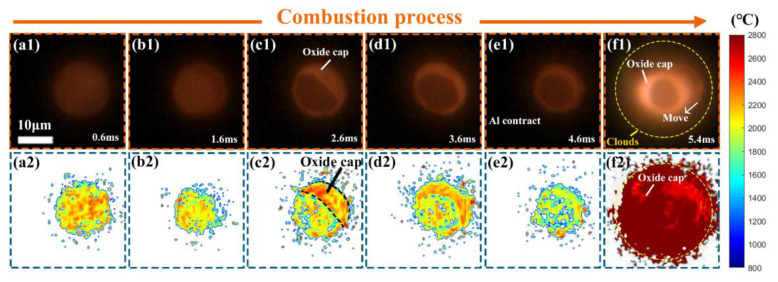
A sequence of two-dimensional flame images and corresponding temperature distribution of the single isolated micro-Al particle burned in air (10,000 fps, exposure time of 99 μs), (**a1**,**a2**) 0.6 ms, (**b1**,**b2**) 1.6 ms, (**c1**,**c2**) 2.6 ms, (**d1**,**d2**) 3.6 ms, (**e1**,**e2**) 4.6 ms, (**f1**,**f2**) 5.4 ms.

A sequence of radiation flame images and corresponding temperature distribution (Figure 5) revealed that the combustion reaction of the micro-Al particle became more vigorous, and the released heat prompted the increase in the radiation temperature of the molten aluminum, the oxide cap, and the clouds. It is worthy to note that the largest flame profile did not exceed the boundary of the original particle, which suggests that the combustion of the micro-Al particle in air belongs to the surface mode and is in agreement with the results in the literature [48,49]. The measured average temperature of ~2200 °C (~2473 K) near the micro-Al particle surface via coupled PMT&CMOS pyrometry is consistent with the gas temperatures (~2450 K) measured by CARS [40], but lower than the flame temperatures (~2600 K) estimated using single-point three-color or two-color pyrometry [50,51].

**Figure 6:**
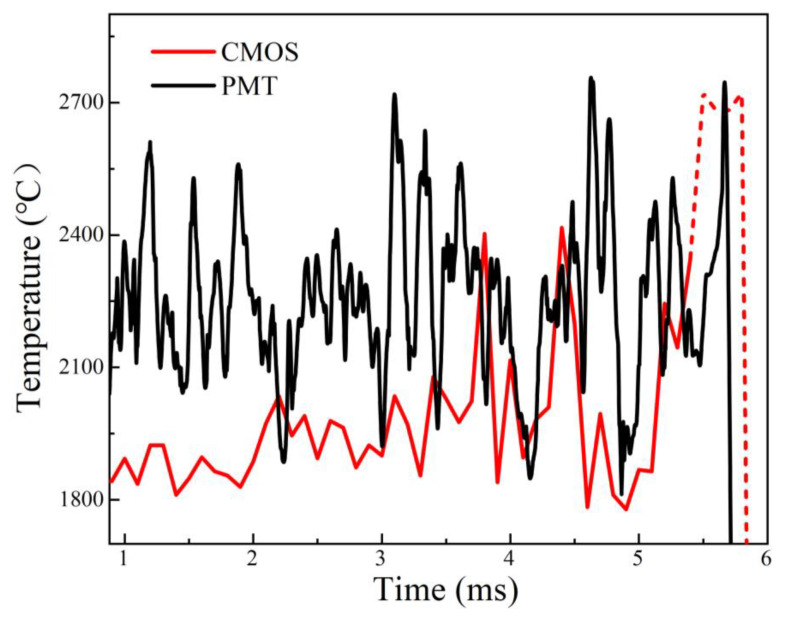
The PMT-based and CMOS-based temperature curves of the single micro-Al particle burned in air via coupled PMT&CMOS pyrometry.

The variation in the highest value in the two-dimensional temperature distribution of the micro-Al particle during combustion is extracted and plotted in Figure 6. Simultaneously, the measured temperature via PMT-based pyrometry is also shown in Figure 6. By comparison, it is found that the two temperature evolutions have similar trends. The CMOS-based temperature at the early stage is obviously lower than the PMT-based one. While at the late stage, the difference in temperature became smaller. It possibly resulted from the radiation difference in the central wavelength of the filters installed in front of the PMTs and CMOS, which can also be testified from the underestimated CMOS-based temperature and the overestimated PMT-based temperature. The underestimation of CMOS-based temperatures is also found in the literature [37] to measure the combustion temperature of Al particles in solid propellant plumes based on imaging pyrometry.

Obviously, the PMT-based pyrometry can obtain the temperature in the whole combustion process, but the CMOS-based pyrometry failed in the end due to overexposure (red dash line in Figure 6). Therefore, the CMOS-based pyrometry faces a problem, which is that it is not capable of performing the whole combustion temperature measurement owing to the large jump in radiation luminance, which can be compensated by the PMT-based pyrometry, testifying the feasibility of coupled PMT&CMOS pyrometry with the combination of single-point temperature and two-dimensional temperature distribution measurement.

#### 3.1.2. Combustion Temperature Measurement of Nano-Al Fuel Aggregates

**Figure 7:**
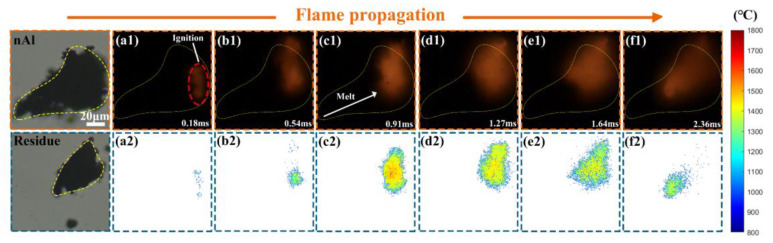
A sequence of two-dimensional flame images and corresponding temperature distribution of a nano-Al aggregate burned in air (11,000 fps, exposure time of 90 μs), (**a1**,**a2**) 0.18 ms, (**b1**,**b2**) 0.54 ms, (**c1**,**c2**) 0.91 ms, (**d1**,**d2**) 1.27 ms, (**e1**,**e2**) 1.64 ms, (**f1**,**f2**) 2.36 ms.

Figure 7 shows a sequence of two-dimensional flame images and the corresponding temperature distribution of a nano-Al aggregate (the equivalent diameter of 56.6 μm). Compared to the micro-Al particle, nano-Al aggregate was locally ignited by a highly focused laser beam, and the flame propagated through the entire aggregate. The combustion of the nano-Al aggregate belongs to the gas-phase combustion mode, since the boiling point of nano-Al is much lower than that of micro-Al particles [30,52]. During combustion, the molten aluminum and the oxide cap could not be clearly observed; only the clouds were recognized. It is significantly different from that of the micro-Al particle.

**Figure 8:**
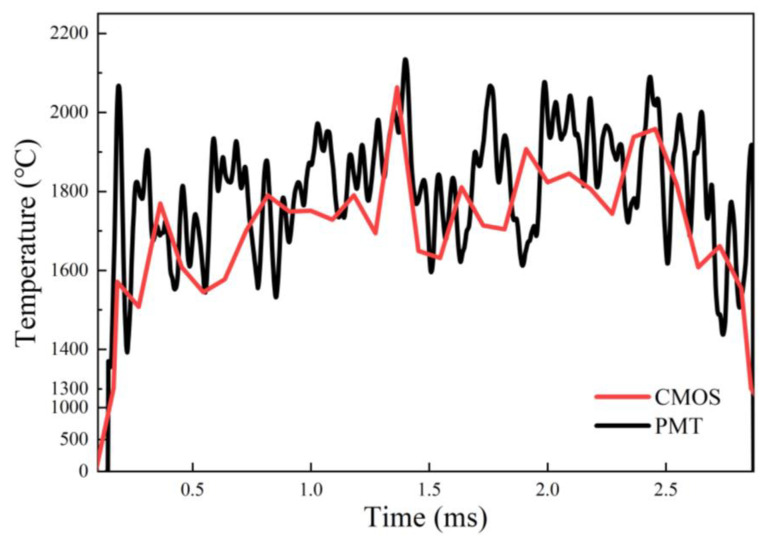
The PMT-based and CMOS-based temperature curves of the nano-Al fuel aggregate burned in air via coupled PMT&CMOS pyrometry.

The combustion temperature evolution of nano-Al fuel aggregate by coupled PMT&CMOS pyrometry is shown in Figure 8. It illustrates that the temperature evolution by PMT kept good consistency with that by CMOS pyrometry. Compared to micro-Al, the highest combustion temperature of nano-Al fuel aggregate was lower ~352.9 °C (CMOS based) and 622.61 °C (PMT based), respectively, suggesting that nano-Al fuel has better ignition performance but less energy release, since the active Al content of nano-Al is significantly lower than that of micro-Al [45].

### 3.2. Combustion Temperature Measurement of Micro-Mg Fuel Particles

**Figure 9:**
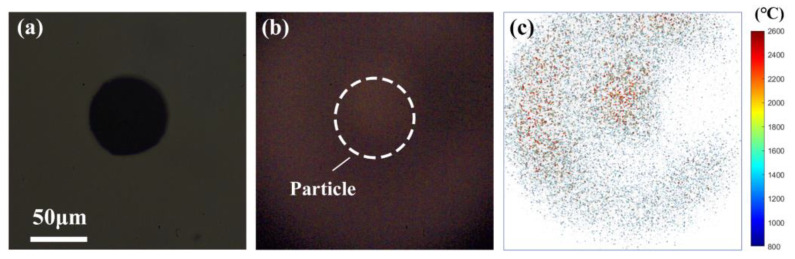
(**a**) The image of a single isolated micro-Mg particle, (**b**) a snapshot of the two-dimensional flame image of the micro-Mg particle burned in air, and (**c**) the corresponding two-dimensional temperature distribution via coupled PMT&CMOS pyrometry (10,000 fps, exposure time of 5 μs).

The combustion of Mg particles is usually attributed to the gas-phase combustion mode because of its low boiling point [41]. Figure 9b shows a snapshot of the combustion flame image of a single isolated micro-Mg particle (Figure 9a), the equivalent diameter of 68.0 μm burned in air. The flame covered the particle, and the flame area was over four times greater than the original particle size, testifying for the gas-phase combustion mode. Correspondingly, the calculated two-dimensional flame temperature (Figure 9c) near the particle surface illustrates that the gaseous flame was mainly composed of the clouds including the quantities of high-temperature MgO particles. In the early stage of the combustion of the micro-Mg particle after ignition, the radiation intensity was so weak, and the ratio of signal to noise was so low, that the two-dimensional flame temperature was difficult to obtain via the CMOS-based pyrometry due to the large calculation error. Therefore, as the combustion reaction proceeded, and the flame radiation intensity in the field of view was high enough, the flame radiation temperature of the micro-Mg particle could be measured by the CMOS-based pyrometry (Figure 9c). It is worthwhile to note that the gas-phase combustion of the micro-Mg particle lasted 1.3 ms, so that only one flame temperature distribution image was acquired.

**Figure 10:**
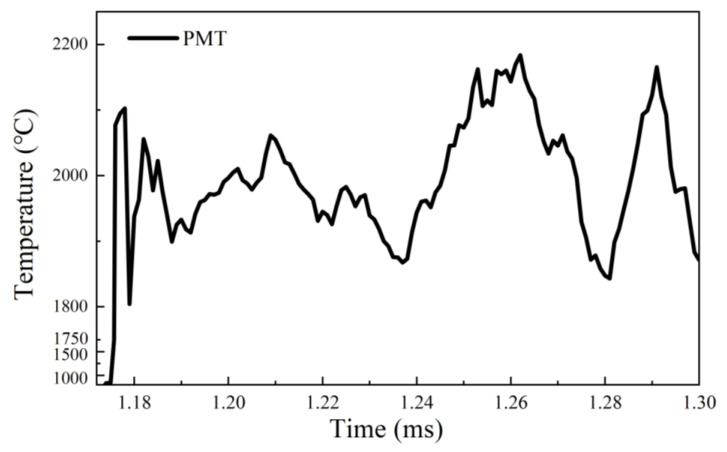
The PMT-based temperature curve of the single isolated micro-Mg particle burned in air via coupled PMT&CMOS pyrometry.

However, the evolving flame temperature was obtained from the PMT-based pyrometry, as shown in Figure 10. The results show that at atmospheric pressure (0.1 MPa), the flame temperature during the combustion of the individual micro-Mg particle repeatedly fluctuated in the range of 1800~2200 °C, over the boiling point of Mg (1090 °C [53]), with multiple peak temperatures of ~2200 °C. The temperatures confirms that the combustion of the micro-Mg particle in air at atmospheric pressure should be dominated by the gas–gas reaction rather than the liquid–gas reaction, which is different from the dominant liquid–gas reaction of Mg and CO_2_ at atmospheric pressure characterized by both homogeneous and heterogeneous combustion reactions [41].

The temperature measurement of the micro-Mg particle via coupled PMT&CMOS pyrometry reveals that the CMOS-based pyrometry meets a challenge for an ultrashort gas-phase burning duration and an extremely low flame radiation luminance, but the PMT-based pyrometry can be adaptable for the case. Therefore, it testifies that the coupled PMT&CMOS pyrometry is a practical and feasible solution to acquire the gas-phase flame temperature of individual particles with a highly spatiotemporal solution.

### 3.3. Combustion Temperature Measurement of Nano-B Fuel Aggregates

**Figure 11:**
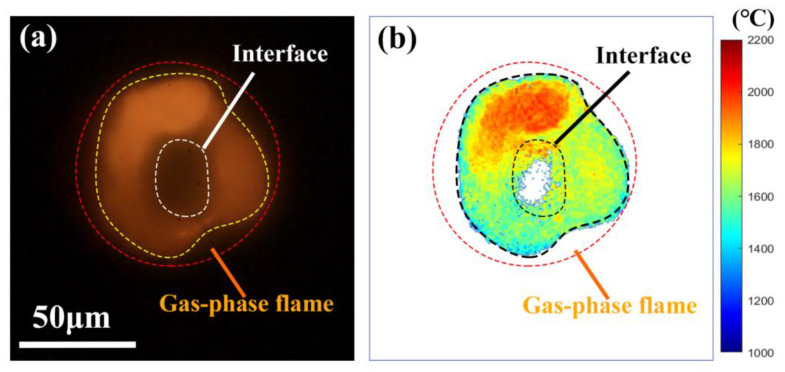
(**a**) A snapshot of the two-dimensional flame image of a single isolated nano-B aggregate burned in air, and (**b**) corresponding two-dimensional temperature distribution via coupled PMT&CMOS pyrometry (10,000 fps, exposure time of 99 μs).

In this work, nano-B fuel aggregates were experimentally investigated to predict the possible application in powder-fueled ramjets. A snapshot of the radiation flame of a single isolated nano-B aggregate (the equivalent diameter of 88.0 μm) burned in air is shown in Figure 11a. It can be clearly observed that the aggregate was surrounded by the gas-phase flame, and an obvious interface existed between the aggregate and the flame. The corresponding two-dimensional temperature distribution (Figure 11b) demonstrated that there were different temperature zones, including the high temperature zone of ~2100 °C at the upper part and the low temperature zone of ~1600 °C at the other parts. The difference of 500 °C shows that the gaseous intermediates did not uniformly diffuse along the radial direction. It indicates that liquid boron oxide (B_2_O_3_) did not uniformly cover the boron particle, resulting in different hinder effects on the oxygen diffusion at different radial directions [54,55].

**Figure 12:**
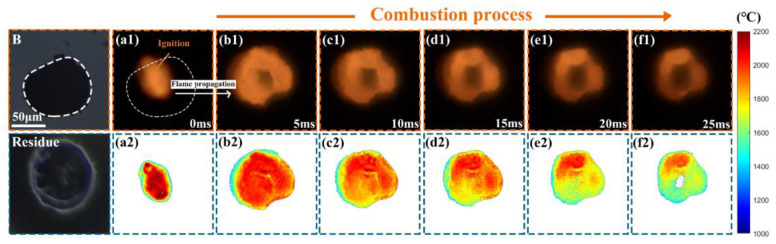
A sequence of two-dimensional flame images and the corresponding temperature distribution of the single isolated nano-B aggregate burned in air (10,000 fps, exposure time of 99 μs), (**a1**,**a2**) 0.0 ms, (**b1**,**b2**) 5.0 ms, (**c1**,**c2**) 10.0 ms, (**d1**,**d2**) 15.0 ms, (**e1**,**e2**) 20.0 ms, (**f1**,**f2**) 25.0 ms.

A sequence of two-dimensional flame images and the corresponding temperature distribution of the nano-B aggregate are shown in Figure 12, which demonstrated that the nano-B aggregate was also locally ignited by a highly focused laser beam, and the flame propagated through the entire particle (Figure 12(a1–b1)) like nano-Al aggregate (Figure 7(a1–f1)), while the flame propagation velocity of the nano-B aggregate was lower than that of the nano-Al aggregate. After that, the combustion of the nano-B aggregate sustained ~20.0 ms, which was longer than that of the nano-Al aggregate (2.18 ms), although the size of the former (88.0 μm) was slightly larger than that of the latter (56.6 μm).

**Figure 13:**
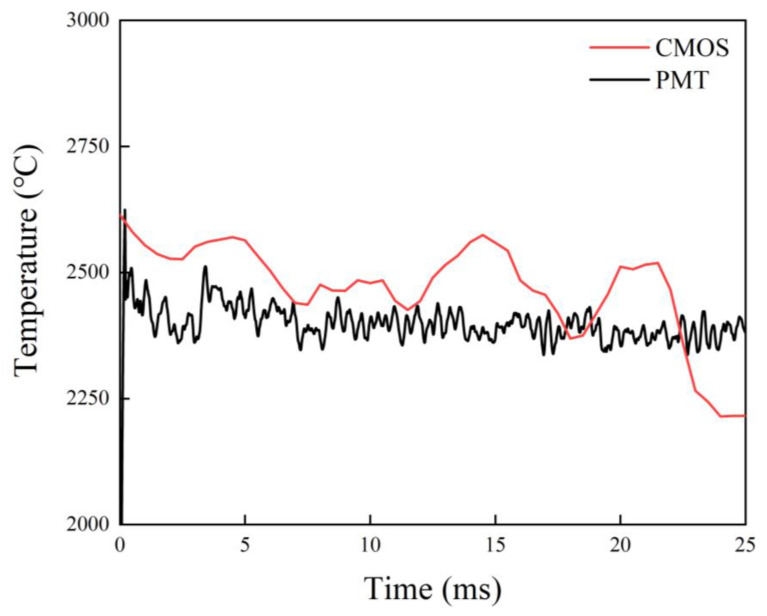
The PMT-based and CMOS-based temperature curves of the single isolated nano-B aggregate burned in air via coupled PMT&CMOS pyrometry.

The whole temperature curves of the nano-B aggregate burned in air measured via coupled PMT&CMOS pyrometry (Figure 13) demonstrated that the combustion temperature slightly oscillated in the range of 2380~2650 °C. Overall, the CMOS-based temperature kept the same pace with the PMT-based temperature, but the former was slightly higher than the latter, especially at the initial stage. It is possibly inferred that at the early stage, liquid boron oxide (B_2_O_3_) produced via the boron reaction with the oxygen was not enough to cover the particle [56].

The average combustion temperature (Figure 13) of the nano-B aggregate burned in air was 600 °C higher than nano-Al aggregate (Figure 8) and micro-Mg particle (Figure 10). It suggests that the burning of B with higher combustion heat (58.8 MJ/kg and 135.2 kJ/cm^3^) released more heat to enhance the combustion temperature, in comparison to nano-Al aggregate and micro-Mg particle. Although, the temperature was slightly higher than that of the micro-Al particle (Figure 6), which means that the heat of the nano-B aggregate was not released completely due to the influence of surface liquid B_2_O_3_.

### 3.4. Combustion Temperature Measurement of micro-B_4_C Fuel Particles

To improve the combustion performance of B fuel particles, boron carbide (B_4_C) based on the modification of B fuel has been widely paid attention to in powder-fueled ramjets because of its high gravimetric combustion heat (52.0 MJ/kg) and volumetric combustion heat (131.0 kJ/cm^3^) [57]. The covalent bonds formed for B_4_C, resulting in producing the gaseous matters CO_2_/CO and providing the pathways to react by diffusing through the liquid oxide layer (B_2_O_3_) to the external surface during oxidation/combustion, accelerating the burning rate [58].

**Figure 14:**
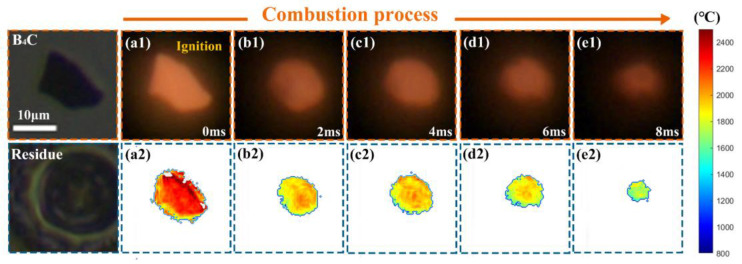
A sequence of snapshots of two-dimensional flame images and the corresponding temperature distribution of a single isolated micro-B_4_C particle burned in air (10,000 fps, exposure time of 50 μs), (**a1**,**a2**) 0.0 ms, (**b1**,**b2**) 2.0 ms, (**c1**,**c2**) 4.0 ms, (**d1**,**d2**) 6.0 ms, (**e1**,**e2**) 8.0 ms.

Figure 14 shows the snapshots of two-dimensional flame images and the corresponding temperature distribution of a single isolated micro-B_4_C particle (the equivalent diameter of 10.9 μm) which burned in air. It demonstrates that the surface of the non-spherical micro-B_4_C particle was fully covered by the flame after ignition, suggesting the surface combustion mode. During combustion, the flame profile of the micro-B_4_C particle became spherical and still covered the particle. Combined with the residue image, it means that micro-B_4_C particle shrank to become spherical. Compared to the annular flame of the nano-B aggregate, the flame of the micro-B_4_C particle was complete and transited from the irregular profile into the nearly spherical profile. It indicated that the liquid oxide layer (B_2_O_3_) did not remarkably hinder the inward and outward diffusion of the gaseous matters of oxidizer and CO_2_/CO.

**Figure 15:**
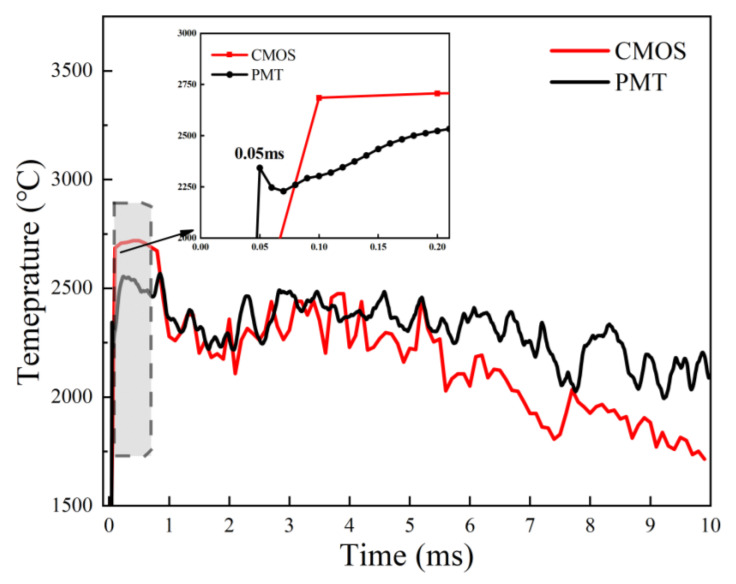
The PMT-based and CMOS-based temperature curves of a single isolated micro-B_4_C particle burned in air via coupled PMT&CMOS pyrometry.

The two evolving temperature curves acquired via coupled PMT&CMOS pyrometry (Figure 15) demonstrated the same trend. The combustion temperature slightly oscillated at about 2400 °C, which is substantially identical to that of the nano-B aggregate. While the size of the micro-B_4_C particle (10.9 μm) is smaller than that of the nano-B aggregate (88.0 μm), which suggests the more excellent combustion performance of the micro-B_4_C particle.

## 4. Conclusions

This work proposed, designed, and manufactured a coupled PMT&CMOS pyrometry with the high temporal and spatial resolution of microsecond and submicron, which could meet the combustion temperature measuring requirements of multiple fuel particles applied in powder-fueled ramjets. Its offline and online calibrations were conducted, and an application of the combustion temperature measuring of five types of fuels was carried out. Through the analysis and discussion of experimental results, some conclusions can be achieved as follows.

(1) The coupled PMT&CMOS pyrometry could mutually testify the feasibility and reliability of the combustion temperature measurement of single metal or composite fuel particles applied in powder-fueled ramjets, overcoming the weakness of independent pyrometry. It could cover the particle size of sub-micron to sub-millimeter with the temporal resolution of a microsecond. The two-dimensional temperature distribution beneficially showed the interfacial combustion characteristics between the particle and flame. The evolving temperature history was helpful to analyze the energy release during combustion and build the combustion models.
(2) Among five types of fuel particles, nano-Al aggregate demonstrated better ignition performance than micro-Al; its ignition was heterogeneous, and the local flame propagated the entire aggregate. Nano-B aggregate presented a similar ignition and flame propagating characteristics with nano-Al aggregate. Micro-B_4_C particle had a shorter ignition delay compared to nano-B aggregate. Micro-Mg particle had the shortest ignition delay time.
(3) The combustion of micro-Al and micro-B_4_C particles in air under atmospheric pressure focused on the surface mode, while those of micro-Mg, nano-B, and nano-Al aggregate presented the gas-phase combustion mode. Nano-Al aggregate had a lower combustion temperature and shorter combustion time in comparison to micro-Al. Nano-B aggregate had a higher combustion temperature than nano-Al aggregate and micro-Mg particle, but incomplete burning and low energy release efficiency due to the inhibition effect of liquid B_2_O_3_ on the diffusion of oxidizer through B surface. The combustion of micro-Mg particle was classically diffusion controlled and had the shortest combustion time and the quickest burning rate.

This work provides a novel tool to measure the combustion temperatures of all kinds of fuel particles that could be possibly applied in powder-fueled ramjets and helpfully gives deep insights into the thermodynamic state based on combustion temperature. In the following works, the combustion temperature measurement of fuel particles burned in air with higher pressures of 3.0 MPa and 7.0 MPa will be conducted to reveal the high-pressure combustion mechanism of fuel particles.

## Data Availability

Data could be available on queries.
